# Evaluation of GammaH2AX in Buccal Cells as a Molecular Biomarker of DNA Damage in Alzheimer’s Disease in the AIBL Study of Ageing

**DOI:** 10.3390/life10080141

**Published:** 2020-08-06

**Authors:** Mohammad Sabbir Siddiqui, Maxime Francois, Stephanie Rainey-Smith, Ralph Martins, Colin L. Masters, David Ames, Christopher C. Rowe, Lance S. Macaulay, Michael F. Fenech, Wayne R. Leifert

**Affiliations:** 1 CSIRO Health and Biosecurity, Molecular Diagnostic Solutions, Adelaide SA5005, Australia; mohammad.siddiqui@mq.edu.au (M.S.S.); maxime.francois@csiro.au (M.F.); lance.macaulay@csiro.au (L.S.M.); Michael.Fenech@unisa.edu.au (M.F.F.); 2 School of Agriculture, Food & Wine, the University of Adelaide, Urrbrae 5064, Australia; 3 School of Biological Sciences, the University of Adelaide, Adelaide SA 5005, Australia; 4 Centre of Excellence for Alzheimer’s Disease Research & Care, School of Medical Sciences, Edith Cowan University, Joondalup 6027, Australia; s.rainey-smith@ecu.edu.au (S.R.-S.); r.martins@ecu.edu.au (R.M.); 5 Sir James McCusker Alzheimer’s Disease Research Unit (Hollywood Private Hospital), Nedlands 6009, Australia; 6 The Florey Institute of Neuroscience and Mental Health, The University of Melbourne, Parkville 3052, Australia; c.masters@unimelb.edu.au; 7 National Ageing Research Institute, Parkville 3052, Australia; dames@unimelb.edu.au; 8 Department of Nuclear Medicine & Centre for PET, Austin Health, Heidelberg 3084, Australia; crowe@unimelb.edu.au

**Keywords:** γH2AX, Alzheimer’s disease, DNA damage, mild cognitive impairment, senescence

## Abstract

In response to double-stranded breaks (DSBs) in chromosomal DNA, H2AX (a member of histone H2A family) becomes phosphorylated to form γH2AX. Although increased levels of γH2AX have been reported in the neuronal nuclei of Alzheimer’s disease (AD) patients, the understanding of γH2AX responses in buccal nuclei of individuals with mild cognitive impairment (MCI) and AD remain unexplored. In the current study, endogenous γH2AX was measured in buccal cell nuclei from MCI (n = 18) or AD (n = 16) patients and in healthy controls (n = 17) using laser scanning cytometry (LSC). The γH2AX level was significantly elevated in nuclei of the AD group compared to the MCI and control group, and there was a concomitant increase in *P*-trend for γH2AX from the control group through MCI to the AD group. Receiver-operating characteristic curves were carried out for different γH2AX parameters; γH2AX in nuclei resulted in the greatest area under the curve value of 0.7794 (*p* = 0.0062) with 75% sensitivity and 70% specificity for the identification of AD patients from control. In addition, nuclear circularity (a measure of irregular nuclear shape) was significantly higher in the buccal cell nuclei from the AD group compared with the MCI and control groups. Additionally, there was a positive correlation between the nuclear circularity and γH2AX signals. The results indicated that increased DNA damage is associated with AD.

## 1. Introduction

Alzheimer’s disease (AD) is a neurodegenerative disease that is characterised clinically by severe memory loss, cognitive deterioration, and behavioural changes [1,2]. AD is the most common cause of dementia in old age, representing approximately 60–80% of all dementia cases [3,4,5]. According to the World Health Organization, 46.8 million people were affected by dementia in the year 2015 [6]. It has been estimated that by the year 2030, 74.7 million people will be affected by AD unless effective interventions are implemented [6]. This increase in the prevalence of AD not only reduces the quality of life, health, and wellbeing of those affected, but also causes a significant financial burden at both the social and economic levels [7].

The classic neuropathological lesions in AD consist of (i) aggregated amyloid plaques containing extracellular hydrophobic deposition of amyloid β peptides (Aβ) in the neuronal body, and (ii) neurofibrillary tangles composed of aggregates of hyperphosphorylated and misfolded tau protein (a microtubule-associated protein) that appear within the neurons [8]. AD patients are usually identified by neuropsychological assessment when the disease has progressed to an advanced stage of cognitive impairment when it is already too late to cure [9,10]. Currently, the ability to detect the early stage of AD and track the different stages of AD progression to guide the choice of therapy is limited. The Mini-Mental State Examination (MMSE) is a validated research-based set of 30 questions assessing memory loss, cognitive decline, and visuospatial and language impairment that is currently used as a standard tool for the clinical diagnosis of AD [11,12]. However, the test lacks accuracy for the diagnosis of AD in living subjects, and diagnostic confirmation can only be achieved post-mortem by the examination of the senile plaques and neurofibrillary tangles in the cerebral tissue [13,14]. The most validated AD disease-related established diagnostic biomarkers are from cerebrospinal fluid (CSF) (aβ1-42, total tau, and phosphorylated tau), structural magnetic resonance imaging (MRI) (e.g., hippocampal volumetry), and amyloid positron emission tomography and fluorodeoxyglucose positron emission tomography imaging [15,16]. Mild cognitive impairment (MCI) is an intermediate state between the cognitive changes of normal ageing and the earliest clinical signs of dementia and is represented as a declining cognition that does not meet the diagnostic criteria of dementia [17]. Individuals affected by MCI have a higher risk of developing AD with an annual conversion rate of approximately 10–15% per year [18,19,20]. Recent evidence indicates that AD is a systemic disorder that can be mirrored by subclinical pathologies in various peripheral tissues other than the brain, thereby rationalising the grounds for investigating cellular biomarkers in peripheral tissues for the diagnosis of MCI/AD risk [21,22,23,24,25]. A recent study has shown that salivary Aβ42 levels can be used to diagnose AD as well as to predict the risk of its future onset [26]. There is a need for non-invasive biomarkers and inexpensive diagnostic approaches with high specificity and sensitivity to identify individuals at increased risk of developing MCI and AD so that early diagnosis and the initiation of preventative therapy is commenced to halt progression to irreversible neurological impairment.

Human buccal mucosa has considerable potential as an easily accessible source of cells that can be collected in a minimally invasive manner. Defects in buccal mucosa cells may reflect systemic changes in pathology in other tissues of ectodermal origin, such as the nervous system [27,28,29]. It has been suggested that the ubiquitous presence and different expression of β-amyloid precursor protein (APP) in the buccal mucosa could be a useful means to estimate the regenerative status of tissue [30]. Accumulation of tau protein in the brain is the major component of neurofibrillary tangles, and is the hallmark of AD pathogenesis [31,32]. The amount of buccal cell tau protein was observed at higher levels in AD subjects and correlated with the levels of tau protein in the CSF [33]. AD is associated with genomic DNA damage, and lack of DNA repair capacity could potentially lead to genomic instability [34,35,36,37,38,39]. 

The buccal micronucleus cytome assay has been developed to score the cytological markers of DNA damage, cell death, and regenerative capacity of buccal mucosa cells [34,40]. Individuals who had just been diagnosed with AD, but had not yet taken medication for their condition, had significantly reduced basal buccal cell frequency compared to unaffected age-matched controls suggesting reduced regenerative capacity. Aneuploidy (abnormal chromosomal number) has been investigated in buccal cells of AD patients in comparison with respective controls, with the results showing a higher aneuploidy level in chromosomes 17 and 21, which are known to encode tau and APP, respectively [34,41,42]. A recent study showed abnormal DNA content (e.g., hyperploidy in nuclei; a marker of aneuploidy) in buccal mucosa cells of AD patients [28]. The same study also demonstrated decreased amount of neutral lipids as measured by Oil Red-O staining in buccal cells from MCI patients [28]. Buccal samples of AD patients were tested for telomere shortening and displayed a significantly shorter telomere length when compared to healthy older controls [43]. A previous study suggested that DNA strand breaks may be increased in lymphocytes of MCI and AD patients [44].

In response to double-stranded breaks (DSBs) in chromosomal DNA, H2AX (a member of histone H2A family and part of the chromatin structure) becomes phosphorylated to form γH2AX [45]. γH2AX has also been found to be increased in neuronal cells of AD and with ageing in lymphocytes [46,47,48]. While H2AX is distributed uniformly throughout chromatin, only H2AX molecules located in close vicinity to DSBs become phosphorylated [45,49,50]. The association of astrocyte degeneration and DNA damage with AD has been elucidated by investigating γH2AX signals in astrocytes from the hippocampus, which is known to be the most vulnerable region affected by AD [46]. The results showed a significantly increased number of γH2AX-immunopositive nuclei in the astrocytes of AD patients in comparison to healthy controls, suggesting that astrocytes may be associated with impaired neuronal function and contribute to the pathogenesis of AD [46]. Additionally, a recent study reported elevated γH2AX levels in the hippocampal tissue of individuals with both AD pathology and clinical dementia than those seen in a normal ageing group [47]. γH2AX has been used as a DSB marker in irradiated human buccal cells and was found to be dose responsive in different buccal cell types [51,52]. However, buccal cell DNA damage involving γH2AX, an important marker of DNA damage and DNA damage response, has not been reported in neurodegenerative disorders such as AD. 

Taken together, the evidence outlined above forms the basis of the hypothesis we tested that buccal cells from individuals with MCI and AD exhibit elevated levels of γH2AX compared to buccal cells from healthy controls. To test this hypothesis, the endogenous levels of γH2AX in buccal cells from participants in the Australian Imaging, Biomarkers and Lifestyle Flagship Study of Ageing (AIBL) who were either healthy controls, MCI cases, or AD cases were measured. An automated laser scanning cytometry (LSC) γH2AX protocol was used to measure multiple parameters (area, integral, MaxPixel) of γH2AX signals, as well as the ploidy and nuclear shapes and senescent cells in thousands of buccal cells per subject.

## 2. Materials and Methods

### 2.1. Human Ethics and Clinical Assessment of the Participants

Approval for the Australian Imaging, Biomarkers and Lifestyle Flagship Study of Ageing (AIBL) was from the institutional ethics committees of Austin Health (Parkville, Vic, Australia), St Vincent’s Health (Fitzroy, Vic, Australia), Hollywood Private Hospital (Nedlands, WA, Australia), Edith Cowan University (Perth, WA, Australia), and CSIRO Australia. All volunteers were informed of the purpose of the study and gave written consent before participating in the study. The demographic and health characteristics of participants included in this study have been well characterised and reported previously [53]. Diagnosis of MCI and AD was performed and confirmed by experienced AIBL clinicians using a battery of neuropsychological tests that were selected on the basis that together covered the main domains of cognition that are affected by AD and other dementias [53]. Data reported in this study are from a total of 51 randomly sub-sampled participants, including: (1) the cognitively healthy control group (n = 17); (2) the MCI group (n = 18) clinically diagnosed with MCI; and the (3) AD group (n = 16) clinically diagnosed with AD. Full blood pathology testing was conducted as described previously [54,55]. There were no blood pathology data available for 10 participants.

### 2.2. Buccal Cell Collection and Microscope Slide Preparation

Prior to buccal cell collection, each participant was first required to rinse their mouth twice with water. Small flat-headed toothbrushes were rotated 20 times against the inner part of the cheeks in a circular motion. Both cheeks were sampled using separate toothbrushes. Heads of the brushes were transferred into a 25 mL tube containing 20 mL of Saccomano’s fixative solution and agitated vigorously to dislodge cells into the solution. Cells were then centrifuged at 1000× *g* for 10 min before discarding and replacing supernatant with fresh 5 mL of buccal cell buffer (10 mM Tris (hydroxymethyl) aminomethane, 0.1 M ethylenediaminetetraacetic acid, 20 mM NaCl, pH 7.0. The cell suspension was drawn up and down five times into a 10 mL syringe using a 21G needle in order to maximise the likelihood of dispersing cell aggregates into a single cell suspension. The cell suspension was then passed through a 100 µm filter in a Swinex filter holder to remove clumps of cells. Cell concentration was assessed using a haemocytometer and cells were then cytocentrifuged for 5 min at 600 rpm onto microscope slides to a final number of 3000 cells per cytospot using a Shandon CytospinVR 4 (ThermoFisher Scientific, Waltham, MA, USA). Slides were washed once with distilled water and air-dried for 1 h and subsequently transferred to ethanol:acetic acid (3:1) fixative for 10 min. The slides were air-dried for 1 h and stored in sealed microscope boxes with desiccant at −80 °C until the staining procedure was performed.

### 2.3. Preparation of Buccal Cells for Immunofluorescence

A circle was drawn around each cytospot using a hydrophobic PAP pen (Dako, Australia) and air-dried at 22 °C for 10 min. Slides were rinsed in Dulbecco’s phosphate-buffered saline (DBPS) for 15 min at 22 °C, incubated in 70% ethanol (4 °C) for 20 min and washed in DPBS for 15 min at 22 °C. Buccal cell cytospots were then treated with 150 µL of prewarmed (37 °C) pepsin solution (containing 750 U/ml of porcine gastric mucosa pepsin) in 0.01 M HCl and then covered with parafilm for 30 min at 37 °C in a humidified box. The slides were then washed twice with DPBS for 5 min. Buccal cells were then permeabilised with 1% Triton X-100 for 15 min at room temperature. Slides were then rinsed three times in DPBS, and a blocking step was performed by incubating cells in 10% goat serum for 1 h at room temperature before being washed once with DPBS. The anti-γH2AX antibody was added to each cytospot at a dilution of 2 µg/mL in DPBS containing 10% goat serum and covered with parafilm overnight at 4 °C in a humidified box. Slides were washed three times in DPBS for 5 min and a secondary antibody Alexa Fluor 488 Goat antimouse IgG was added to each cytospot at 2 µg/mL in DPBS containing 10% FBS and covered with parafilm for 1 h at room temperature. Slides were washed three times in DPBS for 5 min and nuclei were counterstained with 4,6-diamidino-2-phenylindole (DAPI) at a concentration of 1 µg/mL for 10 min at room temperature. The excess DAPI was removed by rinsing the slides with a solution containing 300 mM NaCl and 34 mM sodium citrate. Slides were then mounted with coverslips and DPBS: glycerol (1:1) medium. The edges of coverslips were sealed with nail polish to prevent drying prior to performing laser scanning cytometry.

### 2.4. Laser Scanning Cytometry Measurements of γH2AX

**Figure 1:**
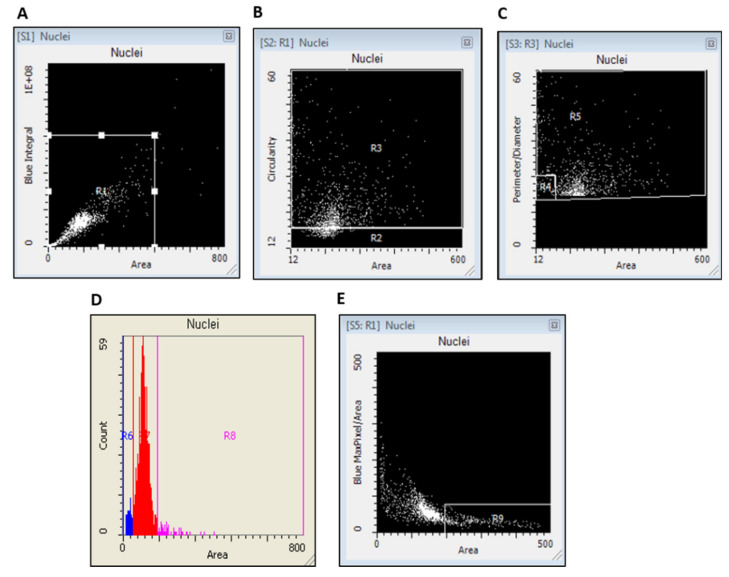
Scattergram and histogram for separation of buccal cell nuclei types by laser scanning cytometry (LSC). A representative example of DNA content scattergram and histogram for a participant from the control group. (**A**) A scattergram was generated to separate cells based on differences in nuclear staining and area by plotting their blue integral versus the area. Nuclei having area values that ranged from 0 to 600 µm^−2^ and blue integral values that ranged from 0 to 4 × 10^7^ (arbitrary units) were separated in Region 1 (R1). (**B**) Nuclei in R1 were analysed by plotting their circularity (*y*-axis) versus nuclear area (*x*-axis) where “round” nuclei were identified in Region 2 (R2). (**C**) Nuclei from Region 3 (R3) were further analysed by plotting their perimeter/diameter ratio (*y*-axis) versus nuclear area (*x*-axis). Two new groups were identified from R3; long nuclei were identified in R4 and oval nuclei in R5. (**D**) A histogram plot of the same data in R1 showing the <2N, 2N, and >2N peaks as represented in R6, R7, and R8, respectively, and the respective frequency of DNA content events scored, showing majority of buccal cells being scored as 2N. (**E**) Nuclei in R1 were plotted against nuclear area versus the ratio of the maximal pixel intensity/area of DAPI fluorescence per nucleus. The cells in R9 had morphometric characteristics of cellular senescence (i.e., increased nuclear size (area) combined with decreased intensity of MaxPixel of DNA-associated fluorescence per nucleus, after DNA staining with DAPI).

Laser scanning cytometry (LSC) measurements were carried out with an iCyte^®^ Automated Imaging Cytometer (Thorlabs, Sterling, VA, USA) with full autofocus function as well as 405 nm and 488 nm lasers for excitation of DAPI and Alexa Fluor 488, respectively. Fluorescence from DAPI (blue) and Alexa Fluor 488 (green) was collected with a photomultiplier tube. Samples were scanned in separate passes (consecutively) to prevent spectral overlap. The nuclei and γH2AX events were contoured using empirically determined thresholds to exclude the scoring of false positives (e.g., small fluorescent debris). The frequency (%) of nuclei containing γH2AX signal was recorded as well as multiple parameters within each nucleus, including the total γH2AX integral (a function of γH2AX intensity and size) and the MaxPixel value (the value of the most intense γH2AX signal/pixel within nuclei). These parameters were generated using the iCyte^®^ 3.4 software and subsequently transferred into excel, then GraphPad Prism for further statistical analyses. Nuclei were also classified into round, long, or oval shapes by utilising the iCyte software parameters which included area, circularity, perimeter, and diameter as described in the legend of Figure 1.

### 2.5. Statistical Analysis

GraphPad Prism 6.01 (GraphPad Prism, San Diego, CA, USA) was used to statistically analyse the data. LSC γH2AX data were checked for normality using the D’Agostino and Pearson omnibus normality test. Differences in relative γH2AX signals in the lymphocytes from control, MCI, and AD groups were compared using the Kruskal–Wallis test for non-Gaussian distributed data followed by Dunn’s multiple comparisons test. Correlation coefficients were obtained using Pearson’s correlation coefficients for Gaussian distributed data and Spearman’s rho for non-Gaussian distributed data. Analysed data are reported as mean ± standard error of the mean (SEM) with *p* < 0.05 considered statistically significant. Receiver-operating characteristic curves (ROC) were prepared for selected γH2AX parameters between the control and MCI or AD groups to obtain the area under the curve (AUC), sensitivity, specificity, confidence interval, and *p*-value.

## 3. Results

### 3.1. Clinical Characteristics of Participants

The mean age, gender distribution (male/female), body mass index (BMI), and MMSE score of AIBL participants in the control, MCI, and AD groups is shown in Table 1. There were no significant differences for gender ratio and BMI between the groups, while there was a significant difference in age (*p* = 0.0039) between control and AD group; however, there was no correlation of age with γH2AX (*r* = 0.08). As expected, there was a significant decrease in the MMSE scores of both the MCI (*p* = 0.0126) and AD (*p* = 0.0001) groups compared with the control group.

### 3.2. Scoring of γH2AX Signals in Buccal Cells by LSC

All nuclei were separated and analysed according to their ploidy status (DNA content) as follows; <2N, 2N, and >2N as well as different nuclear shapes (round nuclei, long nuclei, oval nuclei) and cellular senescence status (see Figure 1). For 2N nuclei, the peak of the nuclei count coincided with the mean DAPI integral.

**Figure 2:**
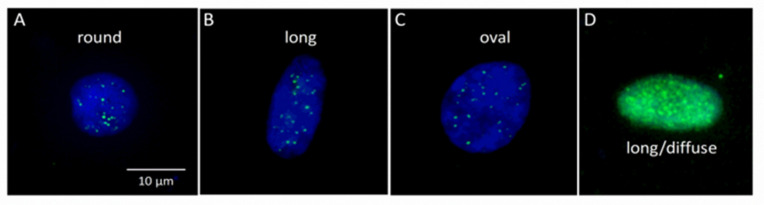
Representative shapes of buccal nuclei and γH2AX foci. Example of buccal cell nuclei visualised (stained with DAPI) with a fluorescence microscope. Nuclei were classified into three categories, i.e., round nuclei (**A**), long nuclei (**B**), and oval nuclei (**C**). Discrete γH2AX foci (green signal) were observed in (**A**–**C**) in these representative images. (**D**) A diffuse γH2AX signal within a nucleus.

**Figure 3:**
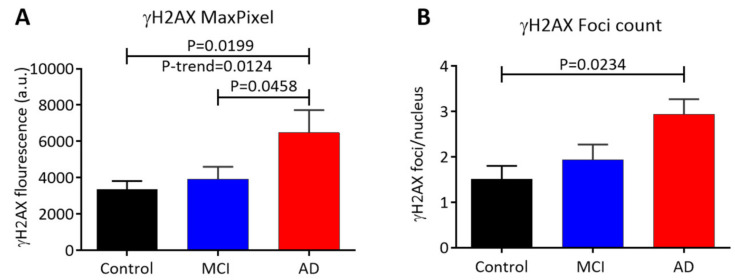
γH2AX MaxPixel and number of foci/nucleus in all cells. (**A**): γH2AX MaxPixel; (**B**): γH2AX foci/nucleus. These parameters were measured by LSC for control (n = 17), MCI (n = 18), and AD (n = 16). Abbreviations: a.u., arbitrary units; AD, Alzheimer’s disease; MCI, mild cognitive impairment. Data are means ± SEM. *p*-values are shown.

Fluorescence images of buccal cell nuclei containing discrete or diffuse γH2AX foci within nuclei were categorised into round, long, and oval nuclei as shown in Figure 2 [51]. Figure 3 summarises the data for the different γH2AX parameters measured (integral, MaxPixel, area, and foci/nucleus) for all nuclei from the control, MCI, and AD groups. Cells were also scored by their ploidy status (i.e., the data for <2N nuclei, 2N nuclei, >2N nuclei, round nuclei, long nuclei, and oval nuclei are shown in Supplementary Table S1). There was a significant increase in the γH2AX integral (*p* = 0.0332) in AD cells compared to control cells in all nuclei (Supplementary Table S1). Consistent with the increase in the γH2AX integral, a significant increase in the γH2AX MaxPixel value (*p* = 0.0199) and the number of γH2AX foci/nucleus (*p* = 0.0234) were also observed in AD cells compared to control cells (Figure 3A,B) and MCI vs. AD (*p* = 0.0458) as shown. Additionally, there was also a significant increase in the linear trend for the γH2AX MaxPixel value (*p* = 0.0124) across the groups (i.e., AD > MCI > control) in all nuclei (Figure 3A). However, there was no significant difference in the area of γH2AX foci (Supplementary Table S1).

The frequency (%) of round, long, and oval shaped nuclei was not significantly different between control, MCI, and AD groups (not shown). Supplementary Table S2 shows a significant increase was observed for the γH2AX integral (*p* = 0.0123), γH2AX MaxPixel (*p* = 0.0014), γH2AX area (*p* = 0.0062), and γH2AX foci/nucleus (*p* = 0.0015) in putative senescent cells when comparing AD versus control cells. The significant increase was also observed for the γH2AX integral (*p* = 0.0349), γH2AX MaxPixel (*p* = 0.0134), and γH2AX area (*p* = 0.0345) in AD senescent cells compared to MCI senescent cells (Supplementary Table S2). There were no differences in the percentage of senescent cells across the groups (Supplementary Table S3).

### 3.3. Nuclear (Morphology Characteristics) Circularity, Integral, and Area in Buccal Cells

**Figure 4:**
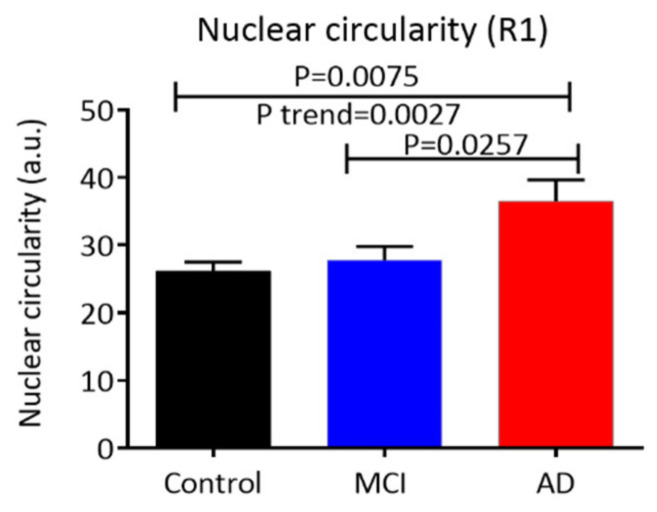
Circularity of buccal cell nuclei. Circularity of all buccal cell nuclei was measured in the control (n = 17), MCI (n = 18), and AD (n = 16) groups. Abbreviations: a.u., arbitrary units; AD, Alzheimer’s disease; MCI, mild cognitive impairment. Data are means ± SEM.

The circularity of buccal cell nuclei in the control, MCI, and AD groups was also measured using the circularity feature available with the iCyte. A high circularity value indicates more irregular shaped nuclei; in contrast, the lowest circularity value indicates a perfect circle. There was a significant increase in nuclear circularity (*p* = 0.0075) in all nuclei of AD cells compared to control cells. In addition, a significant increase in nuclear circularity (*p* = 0.0257) was also observed in AD cells compared to MCI cells. A significant increase in the linear *p*-trend for the nuclear circularity value (*p* = 0.0027) was observed across the groups (i.e., AD > MCI > control) in all nuclei (Figure 4). For the nuclear integral and area, no significant difference was found between the control, MCI, and AD groups.

### 3.4. Receiver-Operating Characteristic Curve

**Figure 5:**
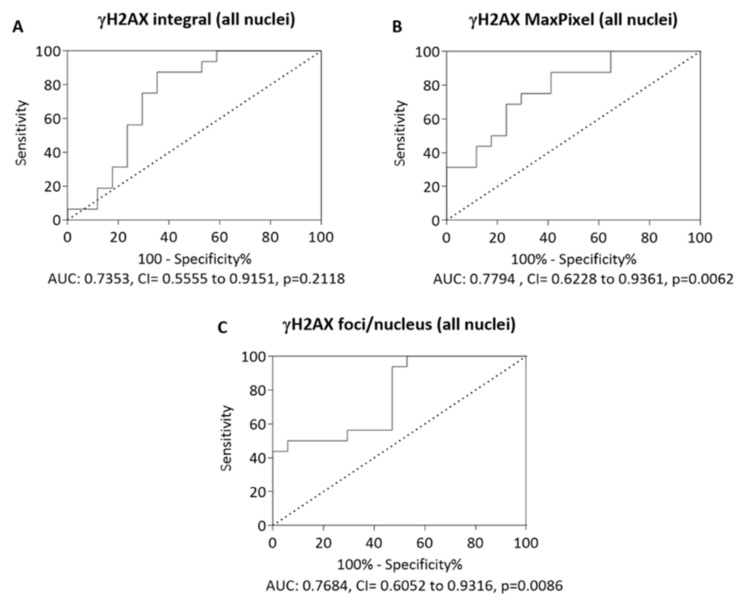
Receiver operating characteristic (ROC) curves for selected LSC-measured γH2AX parameters for control and AD nuclei. ROC curves were generated for the γH2AX integral (**A**), γH2AX MaxPixel (**B**), and γH2AX foci/nucleus (**C**) using measurements in buccal cells from control and AD cells. Abbreviations: AUC (area under the curve), CI (confidence interval).

Since the γH2AX parameters (e.g., integral, γH2AX MaxPixel, γH2AX foci/nucleus) were significantly higher in AD compared to the control group for each category of nuclei, with evaluation of the diagnostic values of these parameters for discriminating AD patients from controls, receiver operating characteristic (ROC) curves were generated. The area under the curve (AUC) values for γH2AX integral, MaxPixel, and foci/nucleus were 0.7353 (*p* = 0.2118), 0.7794 (*p* = 0.0062), and 0.7684 (*p* = 0.0086), respectively (Figure 5A–C). Of all parameters analysed using ROC curves, the γH2AX MaxPixel value showed the greatest value for the identification of AD, with 75% sensitivity and 70% specificity.

### 3.5. Correlation of γH2AX Signals (Integral, MaxPixel) in Different Types of Buccal Cell Nuclei with the MMSE Score

To investigate whether the γH2AX signals in different types of buccal cell nuclei were related to the advancement of cognitive decline in the subjects, the correlations between the γH2AX integral, γH2AX MaxPixel, and MMSE scores were tested. Table 2 summarises the correlation coefficient (*r*) and *p*-values for each of the γH2AX parameters analysed in different types of buccal cell nuclei. The parameters highlighted in bold indicate that the γH2AX integral or MaxPixel negatively correlated with the MMSE score, to varying degrees, dependent on cell type analysed. For example, senescent cells had a strong negative correlation of γH2AX integral with the MMSE score (*r* = −0.5229, *p* = 0.0002).

### 3.6. Correlation of γH2AX Integral with Blood Parameters

Correlation tests were carried out between each of the blood parameters shown in Table 3 and the γH2AX integral values in all nuclei. Of all blood parameters analysed for correlation with γH2AX integral, only total protein was significantly correlated (*r* = 0.332, *p* = 0.0389). In addition, correlation tests were also performed between each of these blood parameters and the γH2AX MaxPixel values. There was no correlation of γH2AX MaxPixel with any blood parameters when data from all nuclei were analysed.

### 3.7. Correlation of γH2AX in Control, MCI and AD Nuclei with Blood Parameters

Correlation tests were carried out between each of these blood parameters and the γH2AX integral or the γH2AX MaxPixel values in control, MCI, and AD nuclei. Table 4 summarises the *r* and *p*-values obtained for γH2AX integral with each of the blood parameters. *p*-values highlighted in bold text indicates significant correlations. Albumin, AP, testosterone, and MCV positively correlated with γH2AX integral (Table 4) in MCI nuclei. Total protein, transferrin, LH, FT4, MCH, and MCHC correlated with γH2AX integral in control nuclei. There was no correlation of any of the blood parameters with γH2AX in the AD group.

## 4. Discussion

The objective of this study was to investigate whether buccal cells from MCI and AD patients have higher levels of endogenous γH2AX (a biomarker of double-strand DNA breaks) compared with healthy controls, with the ultimate aim of testing whether the buccal cell γH2AX assay might be useful as a diagnostic test for those with cognitive impairment and or AD. The γH2AX assay offers an excellent opportunity to robustly measure the levels of DNA double-strand breaks and cellular response in individuals or populations and test its suitability for clinical purposes [56,57,58]. The LSC method was used to quantify endogenous γH2AX in buccal cells from individuals who met the clinical criteria for MCI or AD and in healthy controls. The results of this study showed increased levels of γH2AX (thus DNA damage) in the buccal cells of patients with AD compared to those in cells from MCI patients or healthy controls, and there was a concomitant increase with a linear trend from the control group through MCI to the AD group. This result was further supported by the significantly increased negative correlation between γH2AX signals and MMSE scores when the analysis included all subjects. The LSC protocol developed here simultaneously quantifies different γH2AX parameters (integral, MaxPixel, area, foci/nucleus) in cells with different nuclear DNA content (ploidy status) as well as cells with different morphological features such as nuclear shapes, based on their area, perimeter, diameter, and circularity. Nuclear circularity (irregular nuclear shape) was increased significantly in AD cells compared to control cells and there was a concomitant increase with a linear trend from controls through MCI to AD. A significant positive correlation was also observed between nuclear circularity and γH2AX signals in the different types of nuclei analysed. The results of this study demonstrate that buccal cells exhibit increased levels of endogenous γH2AX in AD cells relative to those from MCI patients and healthy controls, and suggest the possibility of using γH2AX as a potential marker for determining those individuals with MCI that may be progressing to AD.

At present, the analysis of Aβ (1−42), total tau, and phospho-tau-181 in CSF allows reliable, sensitive, and specific diagnosis of AD, but the collection of CSF is an invasive procedure with potential random variation in AD-specific biomarker measurements [59,60,61]. Thus, there is a clear need to search for inexpensive and minimally invasive surrogate markers to diagnose and monitor AD progression. The use of surrogate cells, and particularly exfoliated buccal cells, is of particular interest since buccal cell collection is reliable, fast, relatively simple, cheap, minimally invasive, and painless. Since both the human nervous system and buccal cells are of ectodermal origin, the regenerative potential of the brain, which has been found to be altered in AD, may be mirrored in the buccal mucosa. Studying the buccal mucosa cells from healthy individuals revealed decreased nuclear diameter and cell diameter with increasing age [62]. Another study showed a decrease in the thickness of the epidermis and underlying cell layer with increasing age [63]. It is possible that the lack of regenerative potential of buccal cells from MCI and AD patients may be a consequence of accelerated ageing. A previous study has investigated the formation of micronuclei (a cytogenetic marker of either chromosome segregation or breakage) in buccal mucosa cells. An increased micronuclei frequency was observed in patients with AD compared to age- and gender-matched controls [64]. The same study also reported an abnormal cytome profile characterised by a lower frequency of basal cells, condensed chromatin, and karyorrhectic cells in AD patients, suggesting reduced regenerative capacity in buccal cells from AD patients. Another study showed a significant 1.5-fold increase in trisomy 21 and a significant 1.2-fold increase in trisomy 17 in buccal cells of AD patients compared to matched controls [34], providing further evidence of abnormalities in buccal cells in AD patients. A number of studies have been conducted to assess the association between astrocyte degeneration and DNA damage in AD by investigating the γH2AX signals in astrocytes from the hippocampal region [46,47]. The results from these studies demonstrated increased γH2AX signal in the nuclei of cells from AD patients compared to those from healthy controls. To the best of the researcher’s understanding, there are no earlier reports investigating the levels of γH2AX in buccal cells and their ability to distinguish those individuals with MCI and AD from those of control patients. Since the level of DNA DSBs in buccal cells, as marked by γH2AX immunostaining, has not been previously used to investigate the pathogenesis of AD, the findings from this study support the previous observation of increased γH2AX signals in nuclei of astrocytes from AD patients relative to those of healthy controls [46,47]. In the present study, there was an increasing linear trend in the γH2AX MaxPixel values observed in control through to MCI and AD cells, suggesting that buccal cells from MCI patients may be more susceptible to DNA damage than those from healthy controls. There are no reports investigating γH2AX in buccal cells from MCI patients compared to those from healthy controls; however, the insights from our previous studies carried out in lymphocytes are in line with the observations of the current study, and demonstrate a significant increase in oxidative DNA damage (oxidised DNA bases) in lymphocytes from an MCI group compared with a control group [44]. It is of interest to explore whether MaxPixel γH2AX in AD nuclei represent some unique type of DNA damage (e.g., a site of clustered DSBs).

ROC curve analysis was carried out to assess the diagnostic accuracy of γH2AX assay in identifying individuals with AD from controls. ROC curve for LSC scored γH2AX MaxPixel yielded the area under the ROC curve value of 0.7794 with 75% sensitivity and 70% specificity for the AD (*p* = 0.0062) group, suggesting that measurement of γH2AX MaxPixel in the buccal cell might be useful in discriminating AD and control. Although the good sensitivity and specificity achieved in this study are promising for the value of γH2AX assay in identifying AD from control, given the relatively low number of participants tested within each group, and the lack of defined γH2AX thresholds for determining of test positivity, we cannot currently recommend its routine use in clinical practice. Therefore, it is important to clearly demonstrate its accuracy involving larger numbers of participants tested within each group and standardise the γH2AX assay by validating the stringent cutoff point of test positivity prior to it being widely used routinely for differentiating AD from non-AD and from control.

In this study, irregular nuclear shapes (circularity) were measured using the circularity parameter of LSC in different types of nuclei (e.g., all nuclei, <2N nuclei, 2N nuclei, >2N nuclei). A higher circular value indicates a more irregular nuclear shape, and correspondingly, normal ageing affects nuclear shape that may involve defects in lamins [65]. The results showed a significantly higher circularity in all nuclei of AD cells compared to control cells, as well as in AD cells compared to MCI cells. The higher circularity in AD cells compared to control and MCI cells might be due partly to the accumulation of DNA damage leading to morphometric and cytometric alterations in the buccal mucosa cells of AD patients. Previously, the morphological and cytometric parameters of buccal cells have been assessed using microscopy and ImageJ analysis, respectively, following Papanicolaou staining [66]. The results from that study showed a significant decrease in the number of intermediate buccal cells in the AD group compared to the control group [66]. In addition, evidence of increased levels of DNA damage, indicated by the formation of micronuclei (a biomarker of chromosome mis-segregation) has been previously detected in buccal cells from AD patients and Down syndrome cases who have a high risk of developing AD [64,67]. In our study, the γH2AX integral and MaxPixel values were positively correlated with nuclear circularity in the different types of buccal cell nuclei analysed (data not shown), which may reflect the fact that DNA damage in these cells is associated with an irregular nuclear shape. It is possible that the increased DNA damage in those irregularly shaped nuclei is associated with altered nuclear lamina structure. The nuclear lamina is a filamentous structure under the inner nuclear membrane composed of A-type and B-type lamins [68,69]. Recent studies show that the deficient A-type lamin is associated with altered structural nuclear proteins with a variety of human diseases, including severe premature ageing syndromes. Indeed A-type-lamin-deficient cells have been associated with impaired DNA repair capacity and maintaining telomere localisation, structure, length, and function [70,71]. Moreover, loss of A-type-lamin-leads to localisation of telomeres away from the nuclear membrane towards the center of the nucleus [71]. Colocalisation of γH2AX with a telomere DNA probe allowed visualisation of dysfunctional telomeres [72,73,74]. A previous report in human buccal cells of AD patients showed significantly shortened telomeres in an older AD group in comparison with older controls [43]. Therefore, it is plausible that the positive correlation between nuclear circularity and γH2AX in buccal cells of AD patients observed in this study may be linked with deficient nuclear lamin contributing to telomere dysfunction. Future studies should explore whether the γH2AX signals in buccal cells of AD patients are mostly in the nuclear periphery or aggregated centrally and associated with dysfunctional telomeres which may be due to deficient A-type lamin coupled with increased nuclear circularity. It is possible that irregular nuclear shape caused by a defect in lamins lead to telomere dysfunction and/or shortening. Taken together, altered nuclear morphology, cellular structure, and increased levels of DNA damage associated with dysfunctional telomeres in buccal cells may contribute to the irregular nuclear shape observed in buccal cells of AD patients. A further study of changes in nuclear circularity coupled with multiple DNA damage markers (e.g., γH2AX, 8HOdG) associated with telomere dysfunction and AD-specific markers (e.g., putative tau, Aβ) in buccal cells from a large patient cohort will better assess the likelihood of discriminating AD and MCI patients from healthy controls using these tests.

Cellular senescence is elicited in damaged cells and characterised by the presence of γH2AX, and senescence-associated β-galactosidase (SA-β-gal) activity, and is detectable by immunocytochemistry [75,76]. Previous studies have shown increased number of senescent nuclei during ageing and in age-related diseases [75,76]. It is accepted that older animals exhibit more cellular senescence than younger animals as demonstrated by increased p16 (INK4a), senescence associated β-galactosidase activity, and γH2AX positive signals [73,77,78]. The morphological features of senescent nuclei in cultured fibroblasts after methotrexate (Mtx) treatment have been assessed using the features available in the iCyteR software for LSC [79,80]. In a recent study [79], senescent nuclei were isolated based on the criteria of decreased levels of DAPI staining (MaxPixel staining) paralleled by increases in nuclear size (area) and the simultaneous expression of senescence markers (e.g., the p21WAF1, p16INK4a, or p27KIP1 cyclin kinase inhibitors), and demonstrating that senescent nuclei are flattened and larger in size. To date, the morphological features of senescent nuclei in buccal cells have not been assessed using the features available in LSC. In this study, putative senescent nuclei were identified by plotting the ratio of MaxPixel intensity of DAPI fluorescence per nucleus to nuclear area versus the nuclear size (area). A significant increase in the γH2AX signal was observed in senescent nuclei of AD cells compared to control and MCI cells for all individual γH2AX parameters measured by LSC, suggesting that accumulation of DNA DSBs may contribute to cellular senescence and impaired repairing capacity of senescent nuclei may ultimately contribute to the risk of developing AD. Although previous studies in cultured fibroblasts have characterised the morphological features of senescent nuclei using immunocytochemical analysis of the expression of additional senescent markers, such as the p21WAF1, p16INK4a, or p27KIP1 cyclin kinase inhibitors, our study did not confirm this, but rather attempted for the first time to identify senescent nuclei of control, MCI, and AD cells by their morphometric features alone. It is important to note that senescent cells showed the strongest negative correlation for γH2AX integral and γH2AX MaxPixel in relation to MMSE scores. While investigating the morphological features of senescent buccal cells is important, it is also important for future research to simultaneously measure the expression of senescence markers in conjunction with DNA damage markers (e.g., γH2AX) and AD-specific markers (e.g., aβ1-42, total tau, and phosphorylated-tau) in buccal cells in order to discriminate AD and MCI patients from healthy controls. 

In the present study, from all of the blood parameters examined, only total protein showed a positive correlation with buccal cell γH2AX signals when all samples were analysed together. Correlations between blood parameters and buccal cell γH2AX signals in the control, MCI, and AD groups were further assessed in three separate tests. Although a significant correlation between buccal cell γH2AX signals and several blood parameters (e.g., albumin, total protein, transferrin, FT4, FT3, MCH, MCV) in control and MCI group was observed, in the AD group, no blood parameters showed a significant correlation with buccal cell γH2AX signals. The negative correlation with MCV and MCH are important because these are biomarkers of anemia, which was previously shown to be a risk factor for MCI and AD in the AIBL study [81]. In this study, the positive correlation between transferrin and γH2AX signals suggests that the plasma transferrin levels may have a role in increasing γH2AX signals in AD. However, a previous study showed lower serum transferrin levels in AD patients compared with controls [82]. These results strongly suggest that the development of pathological features of AD is not restricted to the brain but is associated with multiple metabolic changes occurring in peripheral cells [27].

## 5. Conclusions

To date, no studies have assessed the presence of γH2AX in the buccal cells of AD patients relative to control and MCI patients, and the available literature on the use γH2AX as a DNA DSB marker in ageing populations is not yet sufficient to understand the association between DNA DSBs and AD. Identification of reliable biomarkers in non-invasive samples will be useful for early diagnosis and treatment of AD, which may prevent the onset of irreversible AD and reduce the overall economic and human cost of the disease. Buccal cells offer a sample source that is easily obtained in a relatively non-invasive manner. The LSC-based γH2AX protocol may be converted to an ELISA-type format or other simpler analytical technique for cellular γH2AX and therefore may provide a practical tool for assessing DNA DSBs in buccal cells of control, MCI, and AD patients. The levels of γH2AX in buccal cells quantified by LSC may have prognostic implications to understand the pathogenesis of AD better and offer the opportunity to monitor disease progression and the bioefficacy of potential preventative measures (i.e., diet, lifestyle, and therapeutics). Moreover, LSC provides identification and quantification of buccal cell subtypes based on cellular features that were previously not measurable (e.g., nuclear shape, DNA content, nucleus size, nucleus MaxPixel value). Scoring of buccal cell nuclear parameters in conjunction with multiple DNA damage parameters and AD-specific markers will be useful to establish a potential biomarker panel with high specificity for AD patients. Thus, the combination of cytome and proteome approaches to a single sampling of buccal cells may significantly increase the sensitivity and/or specificity for AD diagnosis, which will have relevance not only for future clinical practice but also for the reliable prediction of those individuals who are likely to develop MCI and AD and also to monitor the bioefficacy of a preventative strategy. The buccal cell γH2AX assay may provide a useful method for AD and MCI diagnosis, particularly when sample collection must occur remotely and/or in disadvantaged communities unable to attend more expensive prognostic or diagnostic testing facilities. In this study, a small sample was analysed; therefore, comprehensive studies using large prospective cohorts are warranted in order to validate the suitability of the LSC-based buccal cell γH2AX assay, particularly to identify those in the early stages of AD.

## Figures and Tables

**Table 1:** Clinical characteristics of participants.

	Control n = 18	MCI n = 17	AD n = 16
Sex (M:F)	12:6	11:6	9:7
Age (years)	72.2 ± 1.5	78.7 ± 1.9	81.0 ± 1.8 **
BMI	27.0 ± 1.3	23.4 ± 1.3	24.8 ± 1.1
MMSE score	29.1 ± 0.2	26.0 ± 0.8 *	12.8 ± 1.8 ***

Means and standard error of the mean (SEM) are reported for each group. Significance was accepted at *p* < 0.05. Abbreviations: AD, Alzheimer’s disease; F, female; M, male; MCI, mild cognitive impairment; MMSE, Mini-Mental State Examination score. * *p* < 0.05, ** *p* < 0.01, *** *p* < 0.0001.

**Table 2:** Summary of correlations between LSC scored γH2AX signals vs. MMSE score.

	Parameters	Correlation (*r*)	CI	*p*-Value
All nuclei	γH2AX integral	−0.1899	−0.4014–0.0408	0.0959
	γH2AX MaxPixel	−0.2266	−0.4331–0.0024	**0.0460**
Round	γH2AX integral	−0.3535	−0.5816 to −0.0737	**0.0148**
	γH2AX MaxPixel	−0.4550	−0.6565 to −0.1930	**0.0013**
Long	γH2AX integral	−0.3039	−0.5437 to −0.0183	**0.0378**
	γH2AX MaxPixel	−0.4141	−0.6268 to −0.1440	**0.0038**
Oval	γH2AX integral	−0.3534	−0.5816 to −0.0736	**0.0148**
	γH2AX MaxPixel	−0.4678	−0.6656 to −0.2086	**0.0009**
Senescent	γH2AX integral	−0.5229	−0.7044 to −0.2773	**0.0002**
	γH2AX MaxPixel	−0.5156	−0.6993 to −0.2680	**0.0002**

Parameters highlighted in bold text were considered statistically significant. All are Spearman’s rho correlation. CI: 95% confidence interval.

**Table 3:** Summary of the correlations tested between the γH2AX integral in buccal cells and blood measurements from the Australian Imaging, Biomarkers and Lifestyle Flagship Study of Ageing (AIBL) cohort.

Parameters	Correlation (*r*)	95% Confidence Interval	*p*-Value
Homocysteine	0.0092	−0.1537–0.4472	0.9541
Serum folate	0.1617	−0.377–0.198	0.3125
Vitamin B12	−0.1295	−0.4205–0.1856	0.4195
Red cell folate	0.0005	−0.3151–0.3161	0.9975
Calcium	0.0422	−0.2770–0.3531	0.7985
Cholesterol	−0.0270	−0.1924–0.4290	0.4261
Triglycerides	−0.118	−0.3397–0.2911	0.8704
HDL	−0.1846	−0.4726–0.1391	0.2606
LDL	0.2371	−0.08484–0.5142	0.1461
Albumin	0.0305	−0.2879–0.3428	0.8539
Bilirubin	−0.2013	−0.4860–0.1220	0.2191
Urea	−0.0181	−0.3318–0.2992	0.9131
Creatinine	0.0134	−0.3035–0.3276	0.9354
eGFR	0.0427	−0.2766–0.3535	0.7964
Glucose	−0.2302	−0.5088–0.09207	0.1586
**Total protein**	**0.332**	**0.01837–0.5862**	**0.0389**
ALT	0.0088	−0.3077–0.3234	0.9579
AP	0.0101	−0.3065–0.3247	0.9514
GGT	0.0708	−0.2504–0.3779	0.6684
Ceruloplasmin	−0.2476	−0.5224–0.07374	0.1286
Fe	−0.2834	−0.5498–0.03533	0.0804
Transferrin	0.170	−0.1539–0.4608	0.3009
Trsat	−0.2688	−0.5387–0.05111	0.0980
Ferritin	−0.0201	−0.3336–0.2973	0.9031
Insulin	−0.1066	−0.4084–0.2163	0.5185
Testosterone	0.1546	−0.1692–0.4483	0.3472
LH	0.0245	−0.2933–0.3375	0.8822
FT4	0.1808	−0.1429–0.4696	0.2707
TSH	0.1425	−0.1812–0.4384	0.3868
FT3	0.1999	−0.1234–0.4849	0.2223
Cl	0.04746	−0.2722–0.3577	0.7742
AST	−0.1123	−0.4132–0.2108	0.4961
PCV	−0.0888	−0.3933–0.2334	0.5911
Mg	0.1919	−0.1317–0.4785	0.2418
RCC	−0.0009	−0.3165–0.3147	0.9952
MCV	−0.226	−0.5055–0.09647	0.1665
MCH	−0.2427	−0.5185–0.07897	0.1366
MCHC	−0.1327	−0.4303–0.1909	0.4206
RDW	−0.208	−0.4913–0.1152	0.2039
ESR	−0.1164	−0.4167–0.2068	0.4803
Platelets	−0.05805	−0.3669–0.2623	0.7255
MPV	−0.1251	−0.4239–0.1983	0.4481
WCC	−0.2584	−0.5307–0.06222	0.1122
Neutrophils	−0.2226	−0.5028–0.1001	0.1733
Lymphocytes	−0.1001	−0.4030–0.2225	0.5442
Monocytes	−0.2631	−0.5343–0.05722	0.1056
Eosinophils	−0.1277	−0.4261–0.1958	0.4386
Basophils	−0.2012	−0.4859–0.1222	0.2194

Parameters highlighted in bold text were considered statistically significant. Abbreviations: ALT, alanine aminotransferase; AP, alkaline phosphatase; AST, aspartate aminotransferase; Cl, chloride; eGFR, estimated glomerular filtration rate; ESR, erythrocyte sediment rate; Fe, iron; FT3, free thyroxine; FT4, free triiodothyronine; GGT, gamma-glutamyl transferase; HDL, high-density lipoprotein; LDL, low-density lipoprotein; LH, luteinising hormone; MCH, mean cell haematocrit; MCHC, mean corpuscular haemoglobin concentration; MCV, mean corpuscular volume; Mg, magnesium; MPV, mean platelet volume; PCV, packed cell volume; RCC, red blood cell count; RDW, red cell volume distribution; Trsat, transferrin saturation; TSH, thyroid stimulation hormone; WCC, white cell count.

**Table 4:** Summary of the correlations tested between γH2AX integral scores in buccal cells and blood parameters in the control, MCI, and AD groups from the AIBL cohort.

	Control	MCI	AD
Homocysteine	*r* = −0.070, *p* = 0.804	*r* = 0.514, *p* = 0.106	*r* = −0.175, *p* = 0.518
Serum folate	*r* = 0.193, *p* = 0.491	*r* = 0.256, *p* = 0.448	*r* = 0.134, *p* = 0.635
Vitamin B12	*r* = −0.041, *p* = 0.883	*r* = −0.293, *p* = 0.382	*r* = −0.243, *p* = 0.383
Red cell folate	*r* = 0.288, *p* = 0.299	*r* = 0.003, *p* = 0.993	*r* = −0.149, *p* = 0.595
Calcium	*r* = −0.041, *p* = 0.884	*r* = −0.433, *p* = 0.244	*r* = 0.065, *p* = 0.817
Cholesterol	*r* = 0.467, *p* = 0.079	*r* = −0.279, *p* = 0.467	*r* = −0.072, *p* = 0.799
Triglycerides	*r* = 0.114, *p* = 0.685	*r* = −0.516, *p* = 0.155	*r* = −0.033, *p* = 0.906
HDL	*r* = 0.194, *p* = 0.489	*r* = −0.266, *p* = 0.488	*r* = −0.292, *p* = 0.292
LDL	*r* = 0.465, *p* = 0.080	*r* = −0.016, *p* = 0.968	*r* = 0.292, *p* = 0.802
**Albumin**	*r* = 0.209, *p* = 0.454	***r*** **= 0.724, *p* = 0.027**	*r* = −0.018, *p* = 0.951
Bilirubin	*r* = −0.286, *p* = 0.300	*r* = −0.173, *p* = 0.656	*r* = −0.187, *p* = 0.504
Urea	*r* = 0.500, *p* = 0.058	*r* = −0.181, *p* = 0.640	*r* = −0.326, *p* = 0.236
Creatinine	*r* = −0.276, *p* = 0.320	*r* = 0.407, *p* = 0.277	*r* = −0.038, *p* = 0.893
eGFR	*r* = 0.186, *p* = 0.508	*r* = −0.259, *p* = 0.502	*r* = 0.092, *p* = 0.745
Glucose	*r* = −0.457, *p* = 0.087	*r* = 0.112, *p* = 0.775	*r* = −0.175, *p* = 0.534
**Total protein**	***r*** **= 0.557, *p* = 0.031**	*r* = 0.127, *p* = 0.745	*r* = 0.133, *p* = 0.636
ALT	*r* = −0.224, *p* = 0.421	*r* = 0.109, *p* = 0.779	*r* = −0.035, *p* = 0.901
**AP**	*r* = −0.189, *p* = 0.498	***r*** **= 0.681, *p* = 0.043**	*r* = −0.046, *p* = 0.870
GGT	*r* = −0.108, *p* = 0.700	*r* = −0.087, *p* = 0.824	*r* = 0.025, *p* = 0.931
Ceruloplasmin	*r* = −0.133, *p* = 0.638	*r* = −0.149, *p* = 0.703	*r* = −0.294, *p* = 0.287
Fe	*r* = −0.298, *p* = 0.280	*r* = −0.385, *p* = 0.306	*r* = −0.309, *p* = 0.261
**Transferrin**	***r*** **= 0.628, *p* = 0.012**	*r* = −0.225, *p* = 0.560	*r* = −0.034, *p* = 0.904
Trsat	*r* = −0.344, *p* = 0.209	*r* = −0.294, *p* = 0.442	*r* = −0.282, *p* = 0.308
Ferritin	*r* = −0.252, *p* = 0.366	*r* = 0.025, *p* = 0.949	*r* = −0.100, *p* = 0.721
Insulin	*r* = −0.162, *p* = 0.565	*r* = 0.013, *p* = 0.975	*r* = 0.280, *p* = 0.310
**Testosterone**	*r* = −0.162, *p* = 0.565	***r*** **= 0.684, *p* = 0.042**	*r* = 0.175, *p* = 0.532
**LH**	***r*** **= 0.522, *p* = 0.046**	*r* = −0.235, *p* = 0.542	*r* = −0.177, *p* = 0.527
**FT4**	***r*** **= 0.648, *p* = 0.009**	*r* = −0.078, *p* = 0.842	*r* = 0.155, *p* = 0.582
TSH	*r* = 0.228, *p* = 0.411	*r* = 0.056, *p* = 0.887	*r* = 0.146, *p* = 0.603
FT3	*r* = 0.431, *p* = 0.109	*r* = −0.014, *p* = 0.972	*r* = 0.115, *p* = 0.684
Cl	*r* = −0.173, *p* = 0.650	*r* = −0.269, *p* = 0.485	*r* = 0.173, *p* = 0.538
AST	*r* = −0.173, *p* = 0.536	*r* = 0.032, *p* = 0.935	*r* = −0.185, *p* = 0.508
PCV	*r* = −0.267, *p* = 0.335	*r* = 0.074, *p* = 0.850	*r* = −0.061, *p* = 0.829
Mg	*r* = −0.016, *p* = 0.954	*r* = 0.263, *p* = 0.495	*r* = 0.255, *p* = 0.359
RCC	*r* = −0.081, *p* = 0.773	*r* = 0.279, *p* = 0.467	*r* = −0.071, *p* = 0.799
**MCV**	*r* = −0.425, *p* = 0.115	***r*** **= −0.678, *p* = 0.045**	*r* = −0.045, *p* = 0.871
**MCH**	***r*** **= −0.658, *p* = 0.008**	*r* = −0.657, *p* = 0.055	*r* = 0.054, *p* = 0.848
**MCHC**	***r*** **= −0.689, *p* = 0.005**	*r* = −0.479, *p* = 0.193	*r* = 0.307, *p* = 0.265
RDW	*r* = −0.197, *p* = 0.481	*r* = 0.213, *p* = 0.582	*r* = −0.378, *p* = 0.165
ESR	*r* = −0.157, *p* = 0.577	*r* = −0.209, *p* = 0.589	*r* = −0.186, *p* = 0.507
Platelets	*r* = 0.049, *p* = 0.861	*r* = 0.265, *p* = 0.490	*r* = −0.158, *p* = 0.576
MPV	*r* = 0.057, *p* = 0.844	*r* = −0.143, *p* = 0.713	*r* = −0.438, *p* = 0.103
WCC	*r* = −0.163, *p* = 0.563	*r* = 0.369, *p* = 0.327	*r* = −0.473, *p* = 0.075
Neutrophils	*r* = −0.292, *p* = 0.291	*r* = 0.588, *p* = 0.096	*r* = −0.496, *p* = 0.059
Lymphocytes	*r* = 0.412, *p* = 0.127	*r* = −0.356, *p* = 0.347	*r* = −0.206, *p* = 0.460
Monocytes	*r* = −0.420, *p* = 0.119	*r* = 0.091, *p* = 0.815	*r* = −0.335, *p* = 0.223
Eosinophils	*r* = 0.015, *p* = 0.958	*r* = −0.517, *p* = 0.154	*r* = −0.218, *p* = 0.435
Basophils	*r* = −0.171, *p* = 0.542	*r* = 0.408, *p* = 0.275	*r* = −0.331, *p* = 0.226

Parameters highlighted in bold text were considered statistically significant. Abbreviations: ALT, alanine aminotransferase; AP, alkaline phosphatase; AST, aspartate aminotransferase; Cl, chloride; eGFR, estimated glomerular filtration rate; ESR, erythrocyte sediment rate; Fe, iron; FT3, free thyroxine; FT4, free triiodothyronine; GGT, gamma-glutamyl transferase; HDL, high-density lipoprotein; LDL, low-density lipoprotein; LH, luteinising hormone; MCH, mean cell haematocrit; MCHC, mean corpuscular haemoglobin concentration; MCV, mean corpuscular volume; Mg, magnesium; MPV, mean platelet volume; PCV, packed cell volume; RCC, red blood cell count; RDW, red cell volume distribution; Trsat, transferrin saturation; TSH, thyroid stimulation hormone; WCC, white cell count.

## Supplementary Materials

The following are available online at https://www.mdpi.com/2075-1729/10/8/141/s1, Table S1. Summary of one-way ANOVA tests for different γH2AX parameters measured using LSC in different types of buccal cell nuclei; Table S2. Summary of the one-way ANOVA tests for different γH2AX parameters in putative senescent nuclei; Table S3. Summary of the one-way ANOVA tests for % of senescent nuclei across Control, MCI, and AD.
