# Supplementary material for: Evaluation of GammaH2AX in Buccal Cells as a Molecular Biomarker of DNA Damage in Alzheimer’s Disease in the AIBL Study of Ageing
Source: Life (Basel). 2020 Aug 6;10(8):141. doi: 10.3390/life10080141 (PMC7459751; doi:10.3390/life10080141)
Supplement: Supplementary file 1 [file life-10-00141-s001.pdf]

life

MDPI

Supplementary Table S1. Summary of one-way ANOVA tests for different γH2AX parameters measured using LSC in different types of buccal cell nuclei.

LSC	Con<br>Mean ± SEM	MCI<br>Mean ± SEM	AD<br>Mean ± SEM	Con v MCI<br>p-value	Con vs AD<br>p-value	MCI vs AD<br>p-value
**All nuclei**						
γH2AX integral (×10^6 a.u.)	3.873 ± 1.733	2.280 ± 0.6092	5.088 ± 1.611	NS	**0.0332**	0.0512
γH2AX MaxPixel (a.u.)	3365 ± 458.5	3931 ± 673.5	6477 ± 1244	NS	**0.0199**	**0.0458**
γH2AX area	51.47 ± 21.94	29.34 ± 6.624	51.62 ± 9.776	NS	0.0645	0.1633
γH2AX foci/nucleus	1.510 ± 0.2912	1.943 ± 0.3310	2.940 ± 0.3316	NS	**0.0234**	0.1176
**< 2N nuclei**						
γH2AX integral	2.827 ± 1.105	2.098 ± 0.559	4.253 ± 0.983	NS	**0.0406**	0.0619
γH2AX MaxPixel	3459 ± 476.9	3922 ± 629.8	6498 ± 1263	NS	**0.0216**	0.0745
γH2AX area	40.29 ± 16.22	27.72 ± 5.742	46.54 ± 7.902	NS	**0.0498**	0.1975
γH2AX foci/nucleus	0.8886 ± 0.1652	1.366 ± 0.1802	1.851 ± 0.2257	NS	**0.0064**	0.6317
**2N nuclei**						
γH2AX integral	3.954 ± 1.914	2.201 ± 0.592	5.057 ± 1.788	NS	**0.0485**	0.1273
γH2AX MaxPixel	3309 ± 445.8	3764 ± 625.2	6465 ± 1266	NS	**0.0159**	**0.0281**
γH2AX area	51.56 ± 23.32	29.29 ± 6.655	50.13 ± 10.96	NS	0.1018	0.2603
γH2AX foci/nucleus	1.660 ± 0.3251	2.068 ± 0.3792	3.145 ± 0.3631	NS	**0.0211**	0.1173
**> 2N nuclei**						
γH2AX integral	12.58 ± 6.035	3.842 ± 1.039	18.20 ± 4.103	NS	**0.0069**	**0.0174**
γH2AX MaxPixel	3580 ± 728.9	4879 ± 921.7	8227 ± 1388	NS	**0.0008**	0.0552
γH2AX area	128.8 ± 53.45	52.24 ± 16.46	195.4 ± 40.74	NS	**0.0187**	**0.0414**
**Round nuclei**						
γH2AX integral (x10^6 a.u.)	3.440 ± 1.820	2.182 ± 0.669	4.514 ± 1.722	NS	0.1424	0.4755
γH2AX MaxPixel (a.u.)	3414 ± 449.2	4061 ± 695	6673 ± 1274	NS	**0.0207**	0.0586
γH2AX area	35.76 ± 17.92	22.85 ± 6.45	42.71 ± 10.41	NS	0.2046	0.1749
γH2AX foci/nucleus	1.552 ± 0.287	1.716 ± 0.313	2.934 ± 0.403	NS	**0.0420**	0.0703
**Long nuclei**						
γH2AX integral	2.266 ± 0.9230	2.168 ± 0.6196	4.487 ± 1.253	NS	0.0804	0.2344

LSC	Con<br>Mean ± SEM	MCI<br>Mean ± SEM	AD<br>Mean ± SEM	Con v MCI<br>p-value	Con vs AD<br>p-value	MCI vs AD<br>p-value
γH2AX MaxPixel	3631 ± 586.4	3911 ± 647.2	7692 ± 1437	NS	**0.0119**	0.2690
γH2AX area	35.76 ± 15.19	27.54 ± 6.82	42.02 ± 8.43	NS	0.1659	0.5273
γH2AX foci/nucleus	1.095 ± 0.2044	1.522 ± 0.6077	2.132 ± 0.2940	NS	**0.0209**	0.5141
**Oval nuclei**						
γH2AX integral	3.917 ± 1.734	2.341 ± 0.608	5.407 ± 1.635	>0.9999	**0.0264**	0.0574
γH2AX MaxPixel	3398 ± 495.1	3885 ± 655.5	6366 ± 1123	>0.9999	**0.0135**	0.0527
γH2AX area	56.62 ± 23.98	30.40 ± 6.755	58.62 ± 12.99	>0.9999	0.0535	0.1500
γH2AX foci/nucleus	1.580 ± 0.3293	2.061 ± 0.3624	3.248 ± 0.3491	>0.9999	**0.0091**	0.0862

Parameters highlighted in bold text were considered statistically significant. Data were expressed as mean ± SEM. Abbreviations: a.u., Arbitrary units; AD, Alzheimer's disease; MCI, Mild cognitive impairment; NS = non-significant.

Supplementary Table S2. Summary of the one-way ANOVA tests for different γH2AX parameters in putative senescent nuclei.

Senescent nuclei	Con<br>Mean ± SEM	MCI<br>Mean ± SEM	AD<br>Mean ± SEM	Con vs MCI<br>p-value	Con vs AD<br>p-value	MCI vs AD<br>p-value
γH2AX integral ($\times 10^6$ a.u.)	6.921 ± 2.693	3.590 ± 0.864	12.87 ± 2.87	NS	**0.0123**	**0.0349**
γH2AX MaxPixel (a.u.)	3611 ± 594	4342 ± 734	7613 ± 1058	NS	**0.0014**	**0.0134**
γH2AX area (μm^2)	73.18 ± 27.08	49.93 ± 13.34	152.8 ± 30.1	NS	**0.0062**	**0.0345**
γH2AX foci/nucleus	2.181 ± 0.599	3.635 ± 1.027	5.571 ± 0.671	NS	**0.0015**	0.0761

Parameters highlighted in bold text were considered statistically significant. Data were expressed as mean ± SEM. Abbreviations: a.u., Arbitrary units; AD, Alzheimer's disease; MCI, Mild cognitive impairment; NS = non-significant.

Supplementary Table S3. Summary of the one-way ANOVA tests for % of senescent nuclei across Control, MCI, and AD.

Senescent nuclei	Con<br>Mean ± SEM	MCI<br>Mean ± SEM	AD<br>Mean ± SEM	Con vs MCI<br>p-value	Con vs AD<br>p-value	MCI vs AD<br>p-value
Frequency (%) of cells	14.59 ± 4.047	16.11 ± 4.430	11.13 ± 3.150	NS	NS	NS

Data were expressed as mean ± SEM. Abbreviations: a.u., Arbitrary units; AD, Alzheimer's disease; MCI, Mild cognitive impairment; NS = non-significant.
